# Quantitative global proteome and lysine succinylome analyses provide insights into metabolic regulation and lymph node metastasis in gastric cancer

**DOI:** 10.1038/srep42053

**Published:** 2017-02-06

**Authors:** Yongxi Song, Jun Wang, Zhongyi Cheng, Peng Gao, Jingxu Sun, Xiaowan Chen, Chen Chen, Yunlong Wang, Zhenning Wang

**Affiliations:** 1 Department of Surgical Oncology and General Surgery, The First Hospital of China Medical University, 155 North Nanjing Street, Heping District, Shenyang 110001, China; 2 Jingjie PTM BioLab (Hangzhou) Co. Ltd, No.452, 6th Street, Hangzhou Eco.&Tech. Developmental Area, Hangzhou 310018, China

## Abstract

With the rapid development of high-throughput quantitative proteomic and transcriptomic approaches, the molecular mechanisms of cancers have been comprehensively explored. However, cancer is a multi-dimensional disease with sophisticated regulations, and few studies focus on the crosstalk among multiomics. In order to explore the molecular mechanisms of gastric cancer (GC), particularly in the process of lymph node metastasis (LNM), we investigated dynamic profiling changes as well as crosstalk between long non-coding RNAs (lncRNAs), the proteome, and the lysine succinylome. Our study reports the first qualitative and quantitative profile of lysine succinylation in GC. We identified a novel mechanism through which the TCA cycle and pentose phosphate pathway might be regulated through lysine succinylation in their core enzymes. We then examined the potential of using lysine succinylation as a biomarker for GC and successfully developed a succinylation-dependent antibody for the K569 site in Caldesmon as putative biomarker. Finally, we investigated the relationship between the lysine succinylome and lncRNAs, identifying potential crosstalks between two lncRNAs and one succinylation site. These results expand our understanding of the mechanisms of tumorigenesis and provide new information for the diagnosis and prognosis of GC.

On a global scale, gastric cancer (GC) ranks as one of the most common malignant tumors and one of the leading causes of cancer death, especially in less developed countries^1^. In 2012, there were approximately 951,600 new GC cases and 723,100 associated deaths around the world^1^. GC is a complicated disease due to its histological and etiological heterogeneity^2^ and high tendency for lymph node metastasis (LNM), which remains the most important prognostic factor for GC patients. More than 50% of GC patients have LNM when initially diagnosed, which lead to a 5-year survival rate <30%^3^. Besides, the prognosis of LNM-negative patients was significantly better than that of positive patients^4^. Although the number of metastatic lymph nodes is considered the most significant evaluation of overall survival (OS), the negative lymph node count can play important roles of prognostic evaluation and clinical treatment of GC^5^. Not only in advanced gastric cancer (AGC), LNM in early gastric cancer (EGC) is also a critical factor for assessment of prognosis and therapeutic strategy^6^. Although decreasing trends in GC incidence and mortality rates have been observed in many countries, significant challenges continue to impede our understanding of GC, especially in the process of LNM. Proteomic and protein functional studies have found that numerous protein candidates may play important roles in the LNM of different cancers^7^.

Recently, based on the rapid development of quantitative proteomic approaches by tandem mass spectrometry (MS/MS), differential protein expression levels have been revealed in various tumors^8^,^9^,^10^,^11^,^12^. This may help identify feasible protein candidates as novel diagnostic biomarkers and prognostic targets. Protein post-translational modifications (PTMs) have also been shown to play important roles in regulating protein functions^13^, with specific PTMs on certain substrate residues diversifying and regulating the cellular proteome^14^. Multiple well-known modifications including acetylation^15^ and SUMOylation^16^, as well as several novel PTMs including succinylation^17^ and malonylation^18^, were recently investigated using MS/MS, utilizing high-quality antibodies against PTMs and biochemistry methods. These PTMs widely exist in eukaryotic and prokaryotic organisms^19^. Moreover, PTMs are deeply involved in human diseases^20^ including malignant tumors^21^,^22^, and proteins that mediate epigenetic regulations through histone acetylation and methylation are proven to be associated with the progression of cancers^23^. Furthermore, O-glycosylation changes in the serum of GC patients suggest that it, too, might correlate with GC tumorigenesis^24^.

Among these PTMs, lysine succinylation (Ksucc) is a novel PTM first identified in *Escherichia coli*^17^. As an evolutionarily conserved modification existing in multiple species^25^,^26^,^27^,^28^,^29^,^30^,^31^,^32^, it may affect various cellular functions including the tricarboxylic acid (TCA) cycle and fatty acid metabolism^33^. Therefore, Ksucc may play significant roles in the regulation of cellular metabolism, yet its role in cancer has not been explored. Tumor cells make complicated metabolic alterations characterized by changes in multiple metabolic pathways such as energy production and biosynthetic processes^34^, and protein acetylation, a well-known PTM, is known to regulate metabolism in pancreatic cancer^22^. Based on this, we hypothesized that Ksucc might play a role in cancer by regulating important cellular metabolic pathways. It has been accepted that the pathogenesis of GC involves multi-dimensional aspects due to the complexity and heterogeneity of the disease. Recently, long non-coding RNAs (lncRNAs) were proven to function as potential biomarkers in GC^35^. Interestingly, lncRNAs were reported to link with several protein modifications including acetylation^36^ and phosphorylation^37^, suggesting potential crosstalk between lncRNAs and PTMs.

Here, we have performed multiomics studies to explore the profiling of lncRNAs, the quantitative proteome, and the lysine succinylome in GC for the first time, using the combined advantages of transcriptomics and proteomics. This study, therefore, may increase our understanding of cancer biology and provide a way to screen novel biomarkers and targets for GC diagnostics and treatments.

## Results

### The workflow and strategy for quantitative multiomics in gastric cancer

**Figure 1:**
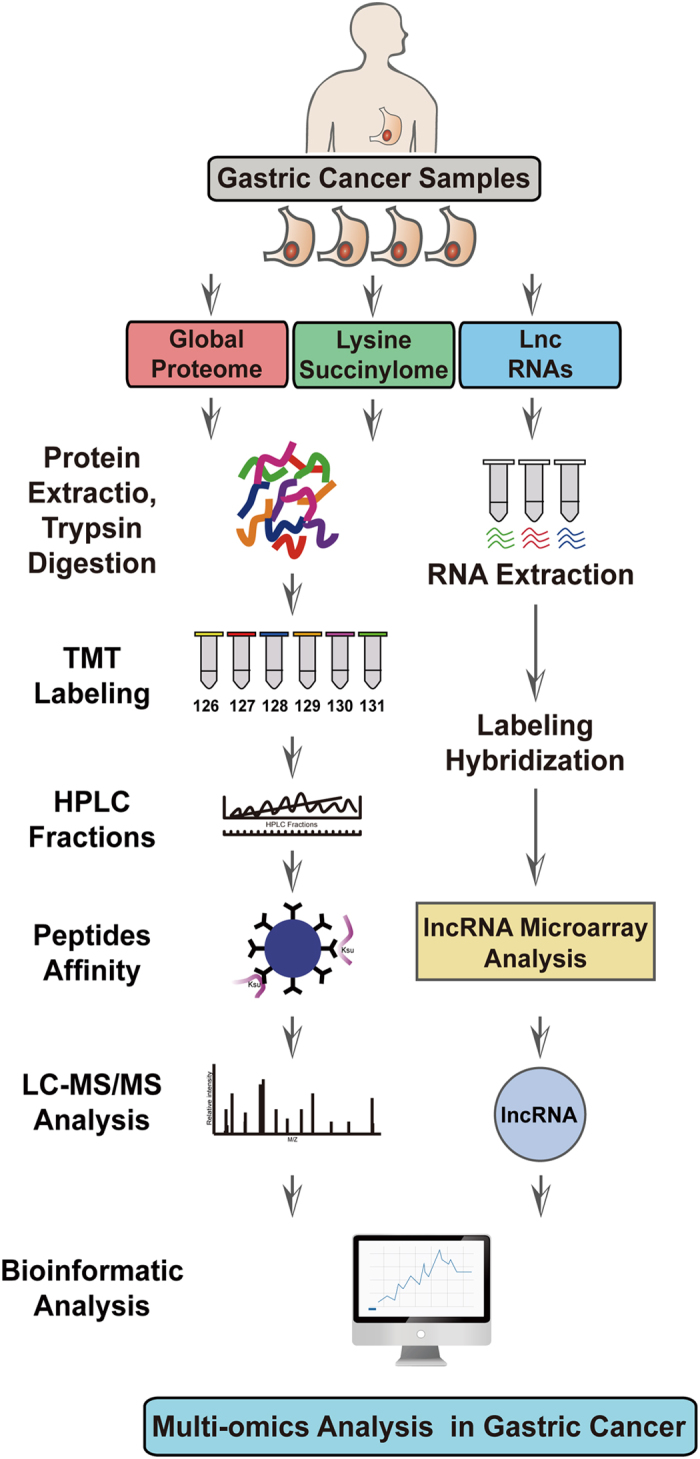
Workflow and strategy for quantitative multiomics in four pairs of gastric cancer tissues and matched normal tissues. LC-MS/MS was used for quantitative proteomic and lysine succinylation analysis, while microarray was performed for global lncRNA profiles.

To comprehensively map the multiomics of gastric cancer (GC), we performed quantitative transcriptomics by microarray and proteomics by LC-MS/MS analyses to explore the profiling of lncRNAs, the global proteome, and the lysine succinylome in four pairs of GC clinical samples. Bioinformatics analyses were employed to make deep multiomics studies in GC. GC clinical samples were obtained via biopsy from patients undergoing surgical resection at the First Hospital of China Medical University (Shenyang, China). Samples included one lymph node-negative case (N0 stage) and three lymph node-positive cases (N1 and N2 stage) according to the 7th edition of the UICC/AJCC TNM staging system. The detailed clinical and pathological data are shown in Supplementary Table 1. The study design and workflow are briefly illustrated in Fig. 1.

### Identification and functional analysis of the global proteome in gastric cancer

To investigate global proteome profiling in GC, we first performed quantitative proteomic studies using TMT labeling, HPLC fractionation, and high-resolution LC-MS/MS analysis (See Supplementary Table 2 for proteomic study summary). Global proteome data generation quality control indicated that proteomics analysis system was robust (See Supplementary Fig. 1).

**Figure 2:**
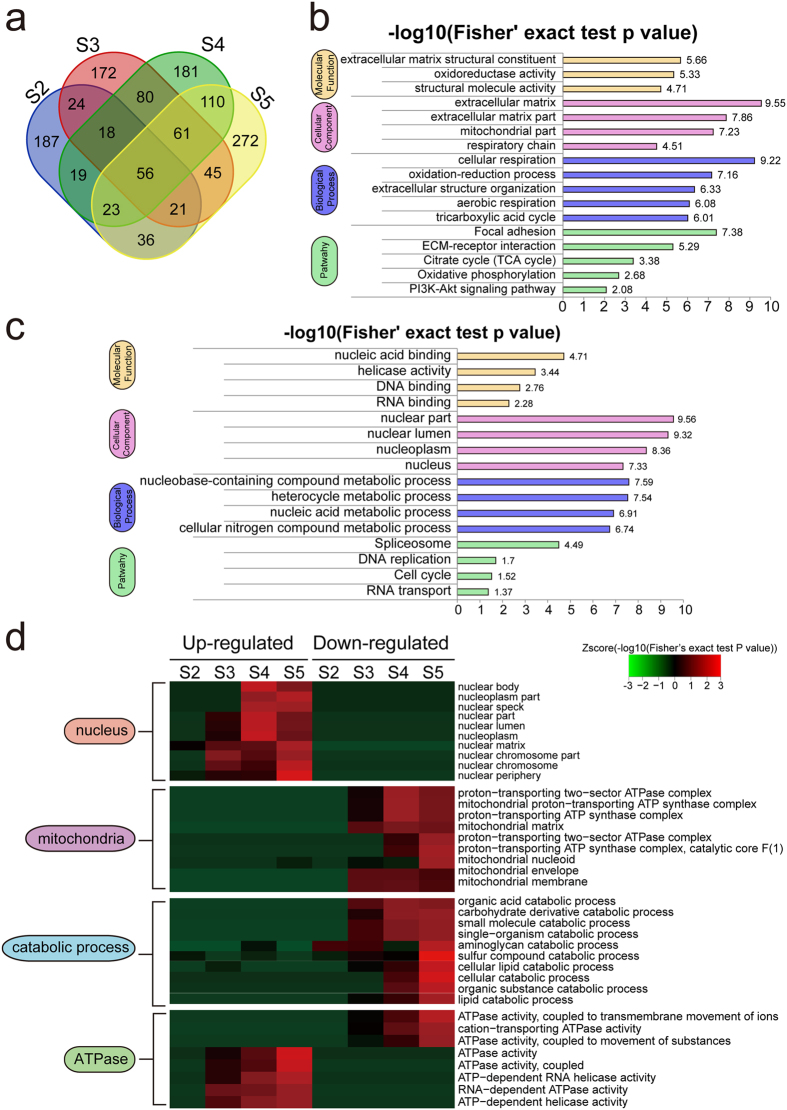
Identification and functional analysis of global proteomics in gastric cancer. (**a**) Venn diagram illustrating differentially expressed proteins in each pair of samples. GO and KEGG pathway functional enrichment of (**b**) down- and (**c**) up-regulated proteins are examined. (**d**) Up- and down-regulated proteins in each pair (tumor vs. normal) are clustered by enriched functional terms discovered in (**b**) and (**c**). Functional enrichment clusters in different biological categories including nucleus, mitochondrion, catabolic process and ATPase are highlighted.

A total of 4,524 proteins were identified from these pairs of GC tissues, among which 3,147 proteins had quantitative changes. Differentially expressed proteins were selected by filtered for an average cut-off change of 1.3-fold^38^,^39^ and p value ≤ 0.05 when comparing tumor samples with their corresponding normal tissues. A Venn diagram illustrating the number of differentially expressed proteins in each pair is shown in Fig. 2a and Supplementary Table 2. In our further analyses, we found the global protein expression profile of four samples (named S2, S3, S4 and S5) were similar despite differences in their clinical and pathological information (Supplementary Fig. 2). We used these four pairs as biological replicates for total-proteomic analysis, aiming to find global differentially expressed proteins. A total of 236 proteins qualified as differentially expressed, including 110 down- and 126 up-regulated proteins. Intensive bioinformatics analyses were then carried out to annotate these differentially expressed proteins (Supplementary Table 2).

Proteins were annotated using Gene Ontology (GO) classification and the KEGG pathway database to further investigate their functions. We then performed enrichment tests to identify GO terms and pathways that were significantly enriched (Fig. 2b and Supplementary Table 2). Many down-regulated proteins were enriched in respiration-related metabolic process terms including oxidation-reduction activity, respiratory chain, and aerobic respiration (Fig. 2b). The KEGG pathway enrichment result was consistent with GO analysis, showing both the TCA cycle and oxidative phosphorylation as negatively enriched (Fig. 2b). The above enrichment results suggested that a hypoxic microenvironment is a central and typical characteristic during cancer development^40^. Other known cancer associated pathways including focal adhesion and the PI3K-Akt signaling pathway^41^,^42^ were enriched in down-regulated proteins. Most of the up-regulated terms were related to nuclear activity including nucleic acid binding, RNA splicing, and DNA replication pathway, suggesting more nuclear activities in GC than in normal cells (Fig. 2c).

As the lymph node statuses differed between the four GC samples (one N0 sample S2, two N1 samples S3 & S4, and one N2 sample S5), we were interested in exploring protein regulation patterns in the process of disease development, especially in lymph node metastasis (LNM) of GC. To do that, we applied GO enrichment based clustering analyses between all four pairs, then classified clusters into several sub-categories based on their functional relevance (Fig. 2d). Consistent with our previous finding that nuclear activities are positively correlated with the development of GC, we found that up-regulated proteins related to the nucleus showed a more obvious enrichment in the process of LNM, while there was no enrichment observed in down-regulated nuclear proteins. On the contrary, in the sub-category of mitochondrion, only down-regulated proteins were significantly enriched, confirming that decreased aerobic respiration is observed in cancer cells. Moreover, down-regulated proteins related to catabolic processes were also obviously enriched in the late stage of LNM. Interestingly, we found ATPase activities to be enriched in both up- and down-regulated proteins in the LNM process. This is not surprising since ATPase proteins take part in multiple essential cellular processes. Up-regulated ATPases included DNA- and RNA-dependent proteins as well as helicase-related APTases involved in nuclear activity such as DNA replication, mRNA processing, and transcription. On the other hand, the down-regulated ATPases were all related to the transmembrane movement of ions and protons that are likely to correlate with oxidative phosphorylation. Taken together, the above clustering results highlight physiological processes which may play important roles in the development of GC, especially in LNM process.

### Dynamic clusters analysis of the protein expression profile in gastric cancer

Protein regulation is likely to be a dynamic process during tumorigenesis as the immune system continuously monitors and fights against cancer progression. As a result, essential proteins likely are initially up-regulated before being inhibited at later stages, as more regulation machineries are involved to control their expression, or vice versa. In order to explore these types of dynamic changes, we ran dynamic clusters analyses based on protein expression quantitation. Compared with traditional single protein analyses, changes in the protein clusters can be utilized to reveal further physiological functions in the dynamic cancer system.

**Figure 3:**
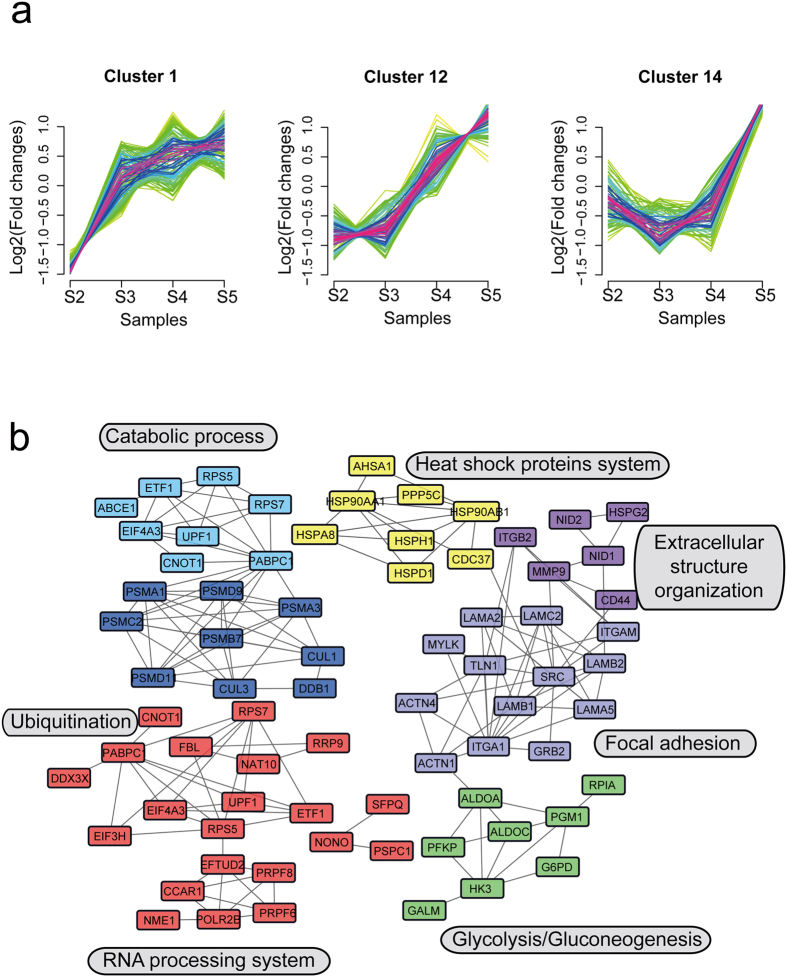
Increased fold-change protein dynamic clusters in gastric cancer development. (**a**) Expression pattern of three increased fold-change protein dynamic clusters. (**b**) The protein-protein interaction network of three increased fold-change protein dynamic clusters. Nodes stand for individual proteins, lines connecting to proteins stand for the correlation between them, and different colors represent different protein functional clusters. Network analyses were performed based on STRING database (http://string-db.org/).

**Figure 4:**
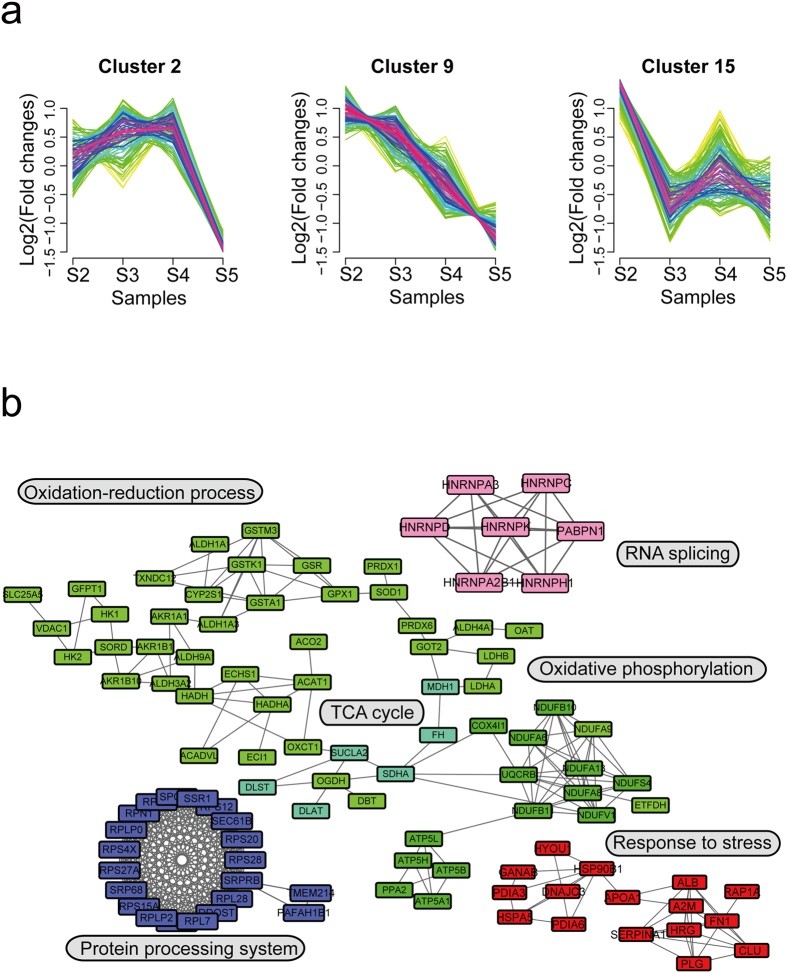
Decreased fold-change protein dynamic clusters in gastric cancer development. (**a**) Expression pattern of three decreased fold-change protein dynamic clusters. (**b**) The protein-protein interaction network of three decreased fold-change protein dynamic clusters. Nodes stand for individual proteins, lines connecting to proteins stand for the correlation between them, and different colors represent different protein functional clusters. Network analyses were performed based on STRING database (http://string-db.org/).

We obtained a series of increased or decreased protein clusters in the process of LNM, with a total of 15 clusters (Supplementary Fig. 3 and Supplementary Table 3) detected. We chose six types of protein clusters for interaction analysis (Figs 3 and 4), aiming to find functionally-related interaction networks from clusters with similar patterns. Clusters #1, #12, and #14 represented a series of proteins whose fold-changes tend to increase as the disease develops (Fig. 3a), while #2, #9 and #15 were protein clusters with decreasing fold-changes (Fig. 4a). We used the STRING database (http://string-db.org/) to analyze correlations between these proteins, and then interconnected them into sub-networks. Proteins in clusters #1, #12 and #14 were involved in biological processes including RNA processing (red), glycolysis/gluconeogenesis (green), focal adhesion (lavender), extracellular structure organization (modena), heat shock proteins system (yellow), ubiquitination (dark blue) and catabolic process (light blue) (Fig. 3b). Proteins in the “fold-change decreased” clusters were likely involved in oxidation-reduction process (green), RNA splicing (pink), protein processing system (blue) and response to stress (red) (Fig. 4b). We also noticed that several significant biological processes or pathways were involved in both increased and decreased protein clusters, such as response to stress, catabolic, and small molecule metabolic process.

### Identification and functional analysis of the lysine succinylome in gastric cancer

As one type of novel discovered PTMs, lysine succinylation (Ksucc) has been proven to be essential for regulating molecular functions such as cellular metabolism in physiological and pathophysiological states^43^. Its metabolic regulation in tumor cells has been intensively studied, as multiple metabolic pathways are altered during tumorigenesis including energy production and biosynthetic processes. However, its roles in cancers have not yet been explored. Protein acetylation, a well-known lysine acylation modification, has been demonstrated to regulate metabolism in pancreatic cancer^22^. We hypothesized that this newly identified lysine acylation type, lysine succinylation, may play a role in cancer by regulating important cellular metabolic pathways.

**Figure 5:**
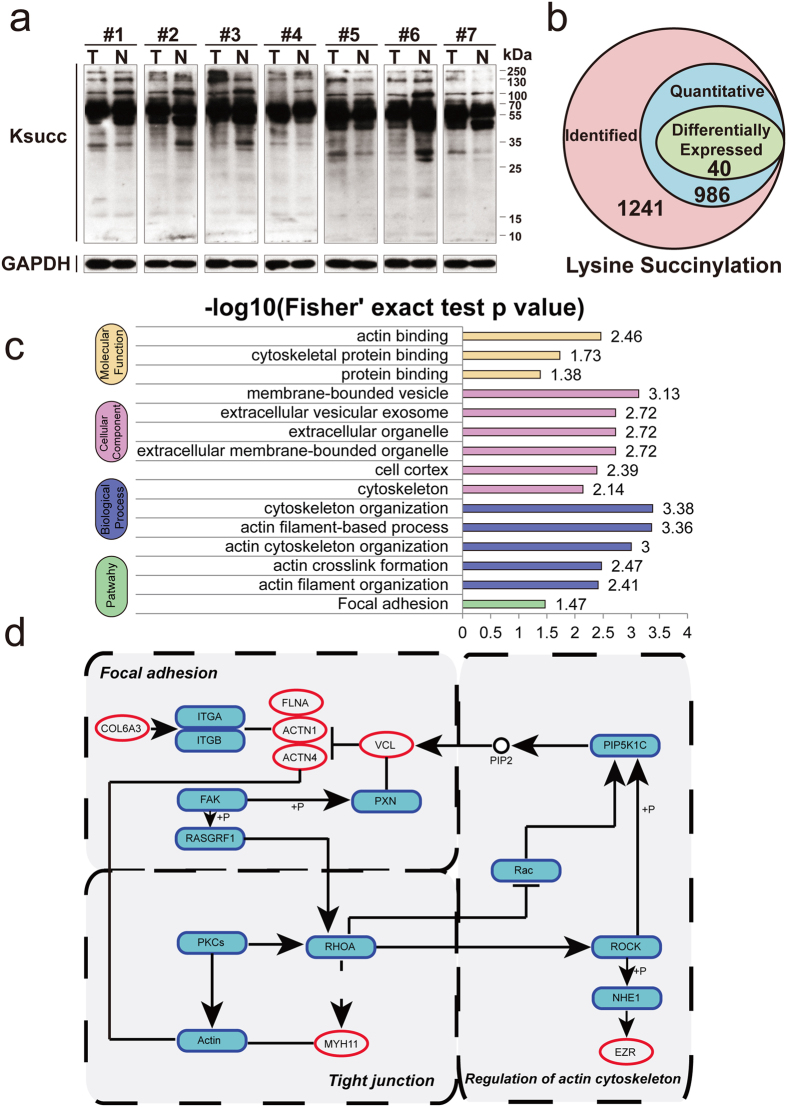
Identification and functional analysis of the lysine succinylome in gastric cancer. (**a**) The expression profile of lysine succinylation in GC tissues by western blotting using a pan anti-succinyl lysine polyclonal antibody (full-length and multiple exposures gels are presented in Supplementary Fig. 4). (**b**) Summary of lysine succinylated sites in GC. (**c**) GO enrichment analysis of down-regulated succinylated proteins. (**d**) Three major pathways (focal adhesion, regulation of actin cytoskeleton and tight junction) of succinylated proteins. Red oval represents lysine succinylated proteins.

First, we tested if the Ksucc levels in GC samples were different from their pairing controls, using western blotting to detect global Ksucc levels. Abundant Ksucc expression levels were observed in GC, with different patterns of Ksucc observed between tumor tissues (designated as T) and normal tissues (designated as N) (Fig. 5a, full-length and multiple exposures gels of global lysine succinylation in Supplementary Fig. 4). To discover differentially regulated Ksucc sites, we applied an integrated quantitative Ksucc approach, using antibody-based affinity enrichment followed by LC-MS/MS analysis. Lysine succinylation data generation quality control indicated that the analysis system was robust (See Supplementary Fig. 5). A total of 1,241 Ksucc sites were identified, among which 986 sites were quantitative (Fig. 5b and Supplementary Table 4).

Among all identified succinylated proteins, approximately 75.8% (640/844) contained only one succinylated site, 15.2% (128/844) possessed two succinylated sites, and 2.5% (21/844) contained five or more Ksucc sites. Notably, six proteins were found to have ten or more Ksucc sites: spectrin alpha chain (17 sites), myosin-11 (12 sites), myosin-9 (13 sites), filamin-A (10 sites), calreticulin (10 sites) and spectrin beta chain (10 sites).

A total of 40 differentially expressed succinylated sites (with a cut-off change of 1.3-fold and p value ≤ 0.05) in 38 proteins were chosen for downstream analyses (see top ten differentially up- and down-regulated Ksucc sites in Table 1). Most of these succinylated proteins were localized in cytoplasm (47%), and 11% of them were distributed in the mitochondria (Supplementary Fig. 6a). As shown in Fig. 5c, down-regulated succinylated proteins were significantly enriched in cytoskeleton associated activities. Differential succinylated proteins in pathways related to cancer metastasis (focal adhesion, regulation of actin cytoskeleton and tight junction) are marked in Fig. 5d. These down-regulated succinylated proteins with functions closely linked with cellular cytoskeleton activities (ezrin (ZER), alpha-actinin-1 (ACTN1), alpha-actinin-4 (ACTN4) and caldesmon (CALD1)) probably contribute to metastasis in GC.

### The potential regulatory roles of lysine succinylation in the TCA cycle and pentose phosphate pathway in gastric cancer

**Figure 6:**
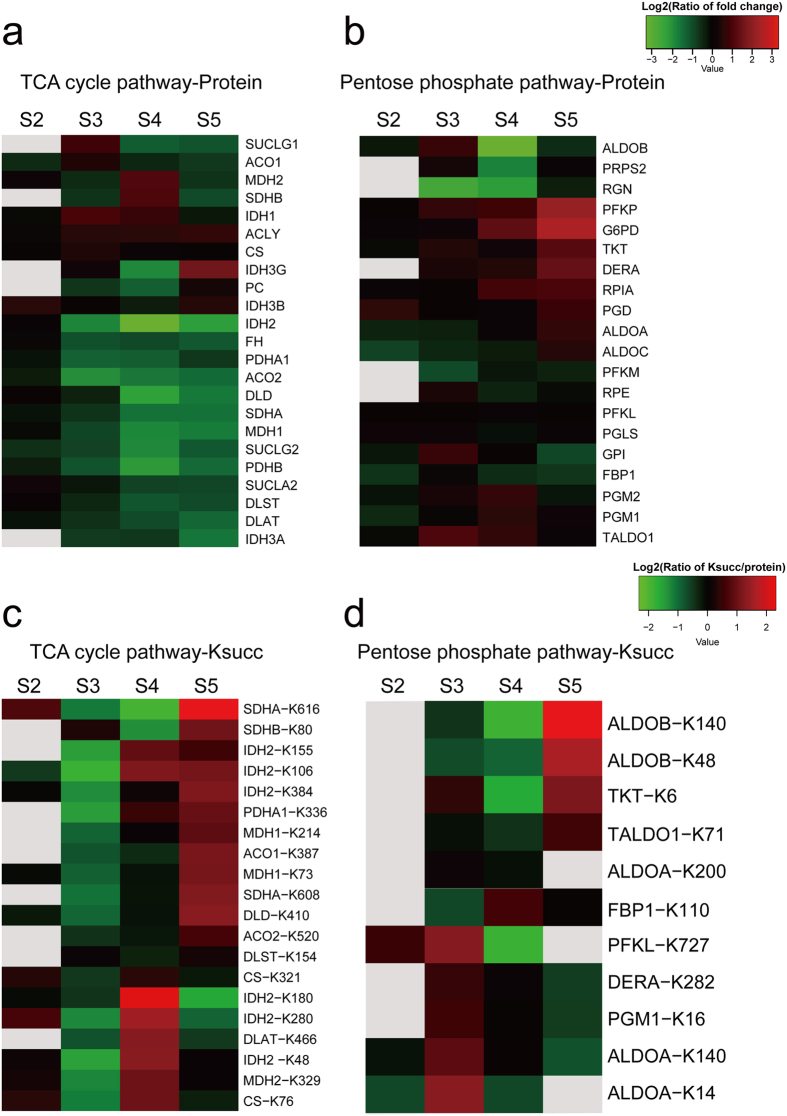
Proteins and Ksucc expression profile in the TCA cycle and pentose phosphate pathway in gastric cancer. (**a**) Protein expression profile in the TCA cycle. (**b**) Proteins expression profile in the pentose phosphate pathway. (**c**) Ksucc expression profile (normalized against protein level) in the TCA cycle. (**d**) Ksucc expression profile (normalized against protein level) in the pentose phosphate pathway. Color gray represents no quantitative data in this specific term.

In the differential expressed protein enrichment analysis, we found that proteins functioning in aerobic respiration processes including the TCA cycle and oxidative phosphorylation were down-regulated in disease development, indicating that aerobic respiration is suppressed in the mitochondria of cancer cells. Ksucc has been shown to be closely associated with the regulation of metabolism, leading us to test whether essential enzymes in cellular respiration have Ksucc modifications in cancer cells. We examined protein and Ksucc expression levels of enzymes in two respiration-related pathways: the TCA cycle and the pentose phosphate pathway (PPP) (Fig. 6 and Supplementary Table 5). The TCA cycle is an essential part of aerobic respiration that occurs in mitochondria. PPP uses the intermediate component from glycolysis to generate the reducing substrate NADPH that is essential for oxidative stress response. The activation of PPP has long been studied in cancer cells^44^,^45^.

As shown in Fig. 6a, TCA cycle-related enzymes decrease expression in the progression of LNM. On the contrary, several proteins in PPP, such as glucose-6-phosphate 1-dehydrogenasepresent (UniProt ID: P11413), 6-phosphofructokinase type C (UniProt ID: Q01813), and deoxyribose-phosphate aldolase (UniProt ID: Q9Y315), are gradually increased in the process of LNM (Fig. 6b).

Interestingly, we found obvious trends of Ksucc expression levels in these two important pathways. Although most of the enzymes in the TCA cycle pathway were gradually decreased, their Ksucc levels (normalized to their own protein level) were increased in the process of LNM (Fig. 6c). Interestingly, enzymes with increased Ksucc sites localized to the second step of PPP (Fig. 6d). Among them, succinylation of the K48 and K140 sites in fructose-bisphosphate aldolase B (UniProt ID: P05062), and the K6 site in transketolase (UniProt ID: P29401) were significantly increased in the N2 stage. Based on above results and previous reported works of Ksucc in metabolism, we believe that lysine succinylation may play significant negative roles in the TCA cycle pathway while playing positive roles in the pentose phosphate pathway.

### Discovery of potential clinical biomarkers of PCNA and lysine succinylation in gastric cancer

**Figure 7:**
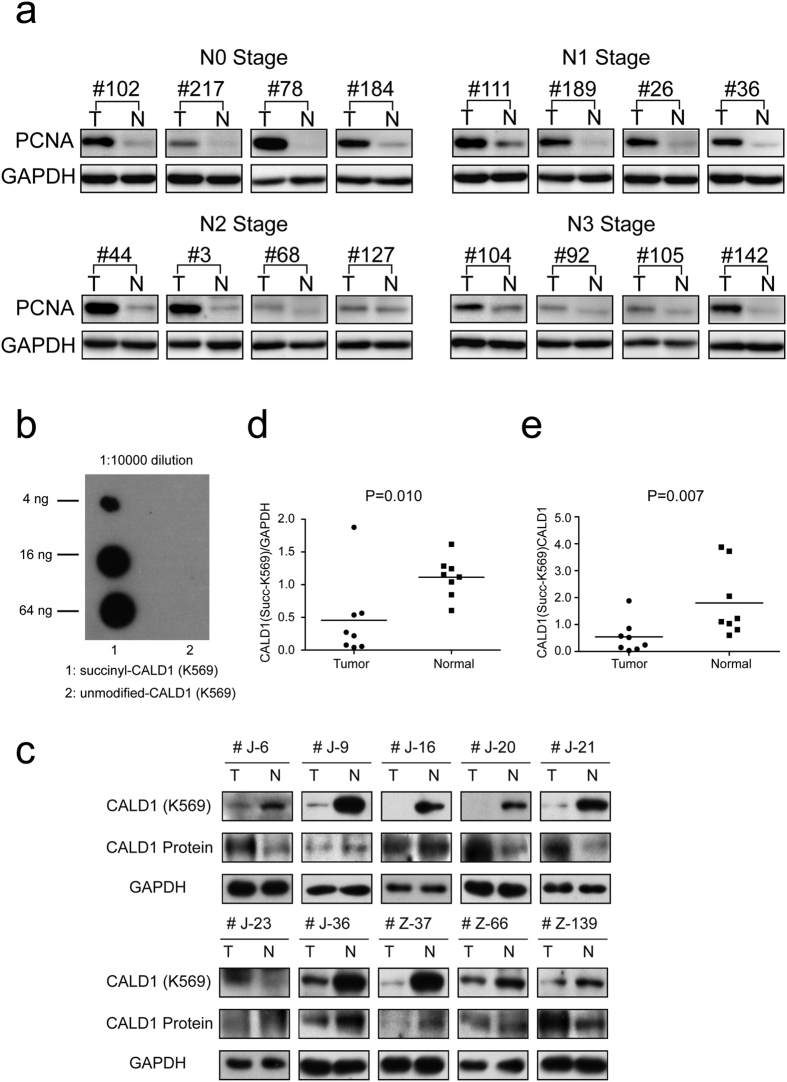
The expression of PCNA and Ksucc-K569 of CALD1 in gastric cancer clinical tissues. (**a**) 16 pairs of GC tissues were involved in the validation experiment by western blotting. We found the expression level of PCNA in cancer tissues was significantly up-regulated when compared with normal ones (see remaining 16 pairs in Supplementary Fig. 7a and full-length gels in Supplementary Fig. 7b). (**b**) Dot blotting analysis on indicated amount of peptides using purified succinyl-CALD1 (K569) antibody diluted 1:10,000. The corresponding peptides and sequences are CLELEEL-(succinyl) K-KKREER and CLELEELKKKREER, respectively. (**c**) The Ksucc expression level of K569 in CALD1 protein shows a significantly decreased level when compared to matched normal tissues by western blotting (full-length gels are presented in Supplementary Fig. 8). (**d**) Quantification of eight pairs show that the decrease of Ksucc-K569 in CALD1 is statistically significant (p = 0.010). (**e**) Quantification shows that Ksucc-K569 is statistically decreased when normalized against its protein level (p = 0.007).

Quantitative proteomic approaches enable us to find potential and feasible protein candidates that may function as novel biomarkers or targets. Proliferating cell nuclear antigen (PCNA) has been shown to be closely associated with several cancers, such as breast^46^, cervical^47^ and gastric cancer^48^. In order to confirm the accuracy of MS/MS data and experimental evidences, we performed validation study of PCNA in other 32 pairs of GC tissues using western blotting analysis (Fig. 7a, see remaining 16 pairs in Supplementary Fig. 7a and full-length gels in Supplementary Fig. 7b). We selected four pairs from N0, N1, N2 and N3 stage, respectively. The expression of PCNA was significantly up-regulated in cancer tissues when compared with their matched normal ones that may reveal its roles of potential biomarker. This result was absolutely coincident with our MS/MS data illustrating the accuracy of our results mentioned above.

In order to explore the possibility of Ksucc being a clinical biomarker in GC, we investigated one top-differentially succinylated protein, caldesmon (CALD1), from the above PTM analysis. CALD1 has been reported to be associated with tumor metastasis^49^ and drug resistance^50^, and its potential role in GC metastasis was illustrated previously^51^. This actin-binding protein functions as a significant regulator of podosome formation, thus regulating cancer cell invasion^52^. Its Ksucc levels are significantly differentially regulated in GC, while its protein expression level is not altered. Thus, we were interested in whether the lysine succinylation of CALD1 can be used as potential biomarker.

We have successfully developed a site-specific primary antibody against succinylation at site K569 of CALD1. The modification specificity of the purified antibody was characterized by ELISA and dot blotting analyses (Fig. 7b). Taking advantage of this site-specific primary antibody, we collected a total of ten pairs of primary human gastric cancer and adjacent normal tissues and detected the level of K569 succinylated CALD1 via western blotting. The results revealed that the succinylation level of CALD1 K569 significantly decreases in GC compared to their matched normal tissues in nine pairs (Fig. 7c). Quantification of eight pairs (excluding two pairs with Ksucc expression levels in tumor tissues too low to be quantified) showed that the decreased level of Ksucc-K569 in CALD1 is statistically significant (p = 0.010) (Fig. 7d). When normalized against its protein level, most pairs of samples also showed a decreased ratio of Ksucc-K569 versus total protein expression of CALD1 in GC (p = 0.007) (Fig. 7e, full-length gels of Ksucc-K569 of CALD1 from ten pairs of GC tissues in Supplementary Fig. 8).

Combined with our MS/MS results, this indicates that Ksucc-K569 of CALD1 may function as a potential biomarker in GC. However, we still need experimental confirmation in more samples to validate this finding.

### Potential crosstalk between lncRNAs and lysine succinylation by co-expression detection and protein-protein interaction analysis in gastric cancer

Previous reports identified that lncRNAs can regulate several protein modifications, including acetylation and phosphorylation. For example, lncRNA-DC, exclusively expressed in human conventional dendritic cells (DCs), was reported to directly bind to STAT3 in the cytoplasm and promoted STAT3 phosphorylation on tyrosine-705^37^. This inspired us to examine relationships between Ksucc and lncRNAs in GC.

In lncRNA microarray analyses, we quantified a total of 11,497 lncRNAs, (Supplementary Table 6). A total of 1,001 lncRNAs were identified to be significantly differentially expressed with fold-change cutoff at 2.0^53^. We established a co-expression patterning-based method to explore the profiling of lncRNAs and Ksucc in GC, and 257 potential co-expressing lncRNA-Ksucc pairs were identified with a co-expression score ≥0.50 (Supplementary Table 6). To further filter the correlation list, we used the STRING interaction database to depict the crosstalks. We defined an association relationship as follows: one succinylated site co-expressing with one lncRNA, and the target protein of the lncRNA interacts with the succinylated protein. We considered that the lncRNA is associated with this succinylated site (Supplementary Fig. 9). With such criteria, we obtained two potential associations of lncRNA-Ksucc with co-expression score ≥0.50 and interaction confidence score ≥0.50. Interestingly, both lncRNA-AX748340 and lncRNA-LOC100507250 negatively associated with the K409 succinylation of delta-1-pyrroline-5-carboxylate synthase (ALDH18A1). Further experiments are required to confirm these two potential correlations between lncRNAs and Ksucc.

## Discussion

In this study, we utilized a combination of quantitative transcriptomic and proteomic approaches to investigate the lncRNAs, global proteins, and post-translational modification profiles in GC samples. Quantitative proteomic analysis showed that proteins with up-regulated expression in tumors are enriched in nuclear activity processes such as cell cycle regulation, DNA replication machinery, and mRNA processing. This result is consistent with our knowledge on physiological changes in tumorigenesis, as deregulated DNA replication in cancers can contribute to genomic instability^54^ and abnormal DNA replication stress is considered a hallmark of cancer that drives cancer development^55^. Functional clustering between samples with different lymph node statuses also revealed that proteins related to the nucleus tend to be up-regulated in disease development and process of LNM. On the other hand, down-regulated proteins were enriched in cellular respiration and mitochondria activity, suggesting that aerobic respiration is inhibited in GC cells.

We did dynamic clustering to identify proteins which change expression patterns during disease progression. In clusters with protein expression tending to increase with the process of LNM, components in ubiquitination system, RNA processing, heat shock protein system, extracellular structure organization, and focal adhesion were enriched. Clusters with proteins whose expression gradually decrease tend to function in aerobic respiration and stress response. This does not completely overlap with functional enrichment results, as dynamic changes of a protein are often more subtle than overall fold changes. However, common terms between these two analyses reveal that genes in TCA cycle and oxidative phosphorylation are not simply inhibited in tumor cells, instead their expression levels tend to decrease as disease becomes more severe. Thus these common genes have potential not only as cancer biomarkers but also as distinguishing factors for lymph node staging.

Succinylation was recently discovered in many eukaryotic organisms as a conserved type of lysine post-translational modification. Several studies suggest that lysine succinylation regulates metabolism, yet the details of these regulations remain elusive. We selected two important glucose metabolic processes (TCA cycle and PPP) that were known to be significantly regulated in cancer cells and detected if their Ksucc level changes follow specific patterns. The TCA cycle is an essential part of cellular respiration downstream of glycolysis that is known to undergo inhibition in cancer, while the PPP is activated to produce ribonucleotides and NADPH to meet the demand of fast growth in cancer cells^45^. Essential enzymes in the TCA cycle or PPP indeed had similar regulation trends in our GC samples compared with reports in the literature. For example, increased expression level and activity of glucose-6-phosphate 1-dehydrogenase (G6PD) are observed in cancer cells^56^, which is consistent with our results. We examined the Ksucc changes in these genes to check for changing patterns. During cancer development, the Ksucc expression levels of enzymes involved in the TCA cycle showed an obvious increasing trend when normalized against dramatically decreasing protein levels, suggesting that Ksucc may play negative roles in TCA cycle enzymes in GC. On the contrary, proteins in PPP are more up-succinylated at later stages of cancers, indicating that activation of lysine succinylation may result in opposite protein expression changes. The TCA cycle generates and consumes succinyl-CoA, an intermediate substrate for lysine succinylation, so we speculate that the balance between succinyl-CoA generation and consumption in metabolic pathways might contribute to this sophisticated regulation of Ksucc and protein expression in the tumor-activated glucose metabolic tuning. Future studies using animal models containing both desuccinylation deficiency (SIRT5KO) and malignant tumors might be able to further study the mechanism of succinylation regulation in tumorigenesis^33^.

By using RNA microarray analyses, differentially expressed lncRNAs were determined between GC tissues and their matched normal tissues. Several known lncRNAs identified in our study, including H19, CCAT1, GAS5 and FER1L4, have previously been recognized to play significant roles in GC development and progression^57^. LncRNAs regulate several protein modifications including acetylation and phosphorylation. In addition, ncRNAs of different origins can regulate histone methylation, resulting in genome regulatory functions^58^. We established an integrated analysis to explore correlations between lncRNAs and lysine succinylation in GC. Surprisingly, only two pairs of lncRNA-Ksucc were identified, and both lncRNAs targeted to a single succinylation site (K409 site of ALDH18A1).

Using the advantages of proteomics approaches, we provide a complete and detailed picture of lncRNA, protein, and lysine succinylation profiles in GC samples. Our study may increase our understanding of tumorigenesis and LNM, especially within energy-related regulation in GC. Moreover, this study provides a novel type of potential biomarker for GC diagnostics.

## Methods

### Gastric Cancer Sample Collection and Preparation

The four pairs of human tissue samples used in our study were obtained from patients diagnosed with GC who underwent surgical resection at the First Hospital of China Medical University (Shenyang, China). The matched adjacent non-tumor tissues were obtained from the furthest part of the resected specimens away from the tumor (>5 cm). The samples were washed with ice-cold phosphate buffered saline (PBS) (GE Healthcare, Beijing, China) to remove residual blood after surgical resection, and then immediately snap-frozen in liquid nitrogen and stored at −80 °C for further analyses. There were no preoperative radiotherapy, chemotherapy, or other therapies done on these four GC patients. This study was conducted according to the principles expressed in the Declaration of Helsinki and was approved by the Research Ethics Committee of China Medical University (Shenyang, China). Informed consents were obtained from all patients. A summary of the clinical and pathological data for these GC patients is shown in Supplementary Table 1. Patients were staged according to the 7th edition of the UICC/AJCC TNM staging system.

### Protein Extraction, Trypsin Digestion, TMT Labeling, HPLC Fractionation and Lysine Succinylated Peptides Affinity Enrichment

GC tissues were firstly grounded into power with liquid nitrogen and then suspended in ice-cold lysis reagent (8 M urea, 5 mM dithiothreitol (DTT), 1% (v/v) protease inhibitor cocktail, 3 μM trichostatin A (TSA), 50 mM nicotinamide (NAM)) with occasional sonication. Cell lysates were centrifuged at 12,000 g at 4 °C for 10 min, and the resulting supernatants were collected. The protein concentration was determined by the 2-D Quant kit (GE Healthcare, USA) according to the manufacturer’s instructions. Afterwards, proteins were precipitated with 15% trichloroacetic acid (TCA) for 4 h at 4 °C and the resulting precipitate was washed for three times with −20 °C acetone. Dried protein pellets were re-suspended in 100 mM tetraethylammonium bromide (TEAB) and digested with trypsin at an enzyme-to-substrate ratio of 1:50 for 12 h at 37 °C. Then the peptides were reduced with DTT and alkylated with iodoacetamide (IAA) in darkness. To ensure complete digestion, the second digestion was conducted by adding trypsin at enzyme-to-substrate ration of 1:100 for 4 h at 37 °C.

Then, tandem-mass-tag (TMT-6 plex) labeling was performed for the quantification of global proteome and lysine succinylome. Four pairs of gastric cancer clinical tissues were involved in our analysis and we divided them into two independent experiments. In the first experiment, the tumor and normal tissues of S2 were labeled with TMT-130 and 131, respectively. In the second experiment, the tumor and normal tissues of S3, S4 and S5 were labeled with TMT-126, 127, 128, 129, 130 and 131, respectively. To lower the complexity of the peptides mixture and enhance the accuracy and throughout of protein identification, the tryptic peptides were fractionated by high pH reverse-phase HPLC using Agilent 300Extend C18 column (5 μm particles, 4.6 mm ID, 250 mm length) with solvent A of 98% H2O and 2% acetonitrile containing 10 mM ammonium formate (pH 10) and solvent B of 2% H2O and 98% acetonitrile containing 10 mM ammonium formate. The LC gradient was from 2% to 60% solvent B for 80 min to generate 80 fractions with 1 min per fraction and then combined into 18 fractions for global proteome and 8 fractions for lysine succylome. The fractionated samples were dried by vacuum centrifugation and stored at −20 °C for further usage.

For lysine succinylome analyses, antibody-based affinity enrichment was performed. Briefly, the tryptic peptides were re-dissolved in NETN buffer (100 mM NaCl, 1 mM ethylene diamine tetraacetic acid (EDTA), 50 mM Tris-HCl, 0.5% NP-40, pH 8.0) and incubated with anti-succinyllysine antibody agarose conjugated beads (PTM Biolabs, Chciago, United States) in a ratio of 15 μL beads/mg protein at 4 °C overnight with gentle shaking. After incubation, the beads were washed four times with NETN buffer followed by twice washes with ddH2O. The bound peptides were eluted with 1% trifluoroacetic acid (TFA) and dried under a vacuum.

### LC-MS/MS Analysis

Peptides were dissolved in solvent A (0.1% formic acid in 2% acetonitrile (ACN), 98% H2O), directly loaded onto a reversed-phase pre-column (Acclaim PepMap100 C18 column, 3 μm, 75 μm × 2 mm, 100 Å, Thermo Scientific). Peptide separation was performed using a reversed-phase analytical column (Acclaim PepMap RSLC C18 column, 50 μm × 15 mm, 2 μm, 100 Å, Thermo Scientific). The gradient was comprised of an increase from 6% to 22% solvent B (0.1% FA in 98% ACN) for 24 min, 22% to 36% for 10 min and climbing to 80% in 3 min then holding at 80% for the last 3 min, at a constant flow rate of 300 nl/min on an EASY-nLC 1000 UPLC system, the resulting peptides were analyzed by Q Exactive Plus hybrid quadrupole-Orbitrap mass spectrometer (Thermo Scientific).

The peptides were subjected to NSI source followed by tandem mass spectrometry (MS/MS) in Q Exactive Plus coupled online to the UPLC. Intact peptides were detected in the Orbitrap at a resolution of 70,000. Peptides were selected for MS/MS using NCE setting as 28; ion fragments were detected in the Orbitrap at a resolution of 17,500. A data-dependent procedure that alternated between one MS scan followed by 20 MS/MS scans was applied for the top 20 precursor ions above a threshold ion count of 1.5E4 in the MS survey scan with 15.0 s dynamic exclusion. The electrospray voltage applied was 2.0 kV. Automatic gain control (AGC) was used to prevent overfilling of the ion trap; 5E4 ions were accumulated for generation of MS/MS spectra. For MS scans, the m/z scan range was 350 to 1600. For TMT quantification, the first mass was set as 100 m/z.

### Quantification Method

Peptide quantitation ratio based on the relative intensities of reporter ions within an MS/MS spectrum. A peptide match will only be used for quantitation if it meets this criterion: Minimum score >20, Maximum expect <0.05, at least homology, unique to one protein hit. For each peptide ratio, a correction factor is applied such that the median of the ratios for all peptide matches that pass the quality tests is unity. The median unique peptide ratio is selected to represent the protein ratio with at least two unique peptide ratios. And the statistic algorithm will give each protein a p value calculated from the peptide ratios which could indicate the accuracy of protein quantitation. And only proteins with p value that < 0.05 were considered accurate quantitation.

### Bioinformatics Analyses

Protein functional annotation, enrichment, motif, enrichment-based clustering, dynamic clustering, protein-protein interaction analysis, and other analyses were used in our study. Detailed descriptions of these analyses are described in Supplementary Information.

### Antibody Generation and Western Blotting

The polyclonal antibody specific for lysine succinylation at K569 of caldesmon (CALD1) was produced in collaboration with PTM BioLab, Inc. Eight week old rabbits were immunized with the succinylated antigen peptide (CLELEEL-(succinyl) K-KKREER) of CALD1. General procedures of antisera screening, purification and antibody characterization were followed. The specificity of the purified antibody against this specific epitope was characterized by ELISA and dot blot analyses.

For western blotting, a total of 20 ug of protein were separated by 10% SDS-PAGE then transferred to a polyvinylidene difluoride (PVDF) or nitrocellulose (NC) membrane. The membrane was blocked with 5% (w/v) skim milk in TBST and incubated overnight at 4 °C with the pan anti-succinyl lysine antibody (1:1000 dilution; PTM Biolabs), anti-GAPDH antibody (1:1000; Cell Signaling Technology), anti-PCNA antibody (1:500; HuaAn Biotechnology, Hangzhou) or anti-Caldesmon antibody (1:1000; Wanleibio, Shenyang). The membrane was then incubated with horseradish peroxidase-conjugated goat anti-rabbit antibody (1:5000; Thermo) for 1 h at room temperature. The membrane was washed with TBST buffer and visualized with enhanced chemiluminescence (ECL) immunoblotting detection reagents (Millipore). Quantitation of western blotting was performed using the measuring function in ImageJ software (http://rsbweb.nih.gov/ij/).

## Additional Information

**How to cite this article**: Song, Y. *et al*. Quantitative global proteome and lysine succinylome analyses provide insights into metabolic regulation and lymph node metastasis in gastric cancer. *Sci. Rep.* **7**, 42053; doi: 10.1038/srep42053 (2017).

**Publisher's note:** Springer Nature remains neutral with regard to jurisdictional claims in published maps and institutional affiliations.

## Supplementary Material

Supplementary Information

Supplementary Table 1

Supplementary Table 2

Supplementary Table 3

Supplementary Table 4

Supplementary Table 5

Supplementary Table 6

## Figures and Tables

**Table 1:** Top ten differentially up- and down-regulated lysine succinylation sites.

Gene Name	UniProt Accession	Ksucc Position	MaxQuant Score	Modified sequence	Log2 C/N Ratio	Regulation
STAT1	P42224	279	93.649	_K(su)LEELEQK_	1.614	up
EPCAM	P16422	129	109.07	_RTDK(su)DTEITCSER_	1.491	up
SFN	P31947	77	120.63	_SNEEGSEEK(su)GPEVR_	1.198	up
OAS2	P29728	239	63.091	_K(su)DNFDIAEGVR_	1.054	up
GBP1	P32455	487	63.624	_EK(su)EIEVER_	1.051	up
ALDH18A1	P54886	409	72.34	_K(su)DLEEAEGR_	0.899	up
POR	P16435	291	56.916	_K(su)LNQGTER_	0.816	up
EEF1G	P26641	243	63.565	_QK(su)PQAER_	0.787	up
HSP90AB1	P08238	550	112.44	_EGLELPEDEEEK(su)K_	0.644	up
CALD1	Q05682	569	128.06	_QQEAALELEELK(su)K_	−1.112	down
DYNC1H1	Q14204	3284	124.29	_EDLDK(su)VEPAVIEAQNAVK_	−1.184	down
ANXA6	P08133	478	119.39	_AINEAYK(su)EDYHK_	−1.210	down
TXNL1	O43396	226	120.33	_SEPTQALELTEDDIK(su)EDGIVPLR_	−1.244	down
FLNA	P21333	865	66.989	_VK(su)VEPSHDASK_	−1.312	down
COL6A3	P12111	869	119.96	_IIDELNVK(su)PEGTR_	−1.427	down
TAGLN	Q01995	21	144.65	_K(su)YDEELEER_	−1.662	down
VCL	P18206	387	90.475	_K(su)IDAAQNWLADPNGGPEGEEQIR_	−1.809	down
HSP90B1	P14625	800	98.582	_ESTAEK(su)DEL_	−2.170	down
MYH11	P35749	1499	117	_ALEEALEAK(su)EELER_	−2.437	down
