# Supplementary material for: Quantitative global proteome and lysine succinylome analyses provide insights into metabolic regulation and lymph node metastasis in gastric cancer
Source: Sci Rep. 2017 Feb 6;7:42053. doi: 10.1038/srep42053 (PMC5292683; doi:10.1038/srep42053)

**Supplementary Information**

**Quantitative global proteome and lysine succinylome analyses provide insights into metabolic regulation and lymph node metastasis in gastric cancer**

Yongxi Song ^1,#^, Jun Wang ^1,#^ , Zhongyi Cheng ^2^, Peng Gao ^1^, Jingxu Sun ^1^, Xiaowan Chen ^1^, Chen Chen ^2^, Yunlong Wang ^2^ and Zhenning Wang ^1^*

^1^ Department of Surgical Oncology and General Surgery, The First Hospital of China Medical University, 155 North Nanjing Street, Heping District, Shenyang 110001, China

^2^ Jingjie PTM BioLab (Hangzhou) Co. Ltd, No.452, 6th Street, Hangzhou Eco.&Tech. Developmental Area, Hangzhou 310018, China

^#^ These authors contributed equally to this work.

*Correspondence to: Zhenning Wang, Department of Surgical Oncology and General Surgery, The First Hospital of China Medical University, 155 North Nanjing Street, Heping District, Shenyang 110001, China. Tel: +86-24-8328-3556; Fax: +86-24-2270-3578; E-Mail: josieon826@sina.cn.

**Supplementary Methods**

**Global Proteome and Lysine Succinylome Database Searching.**

MS/MS raw data was transformed with Proteome Discoverer (v.1.3.0). For global proteome, the resulting MS/MS data were processed using Mascot search engine (v.2.3.0). Tandem mass spectra were searched against Swiss-Prot human database. Trypsin/P was specified as cleavage enzyme allowing up to 2 missing cleavages. Mass error was set to 10 ppm for precursor ions and 0.02 Da for fragment ions. Carbamidomethyl on Cys was specified as fixed modification and oxidation on Met was specified as variable modifications. For protein quantification, TMT-6plex was selected. False discovery rate (FDR) for peptide was adjusted to < 1% and peptide ion score was set > 20.

For Ksucc peptides, the resulting MS/MS data was processed using MaxQuant with integrated Andromeda search engine (v.1.4.1.2). Tandem mass spectra were searched against Swiss-Prot human database concatenated with reverse decoy database. Trypsin/P was specified as cleavage enzyme allowing up to 3 missing cleavages. Mass error was set to 10 ppm for precursor ions and 0.02 Da for fragment ions. Carbamidomethylation on Cys was specified as fixed modification and oxidation on Met, succinylation on lysine were specified as variable modifications. FDR for peptide was specified at 1%. Minimum peptide length was set at 7 and peptide ions score was set > 40. The site localization probability was set as > 0.75.

The mass spectrometry proteomics data have been deposited to the ProteomeXchange Consortium via the PRIDE partner repository with the dataset identifier PXD004037. Reviewer account details: Username: reviewer36804@ebi.ac.uk. Password: zlpOGrp1.

**Protein Functional Annotation.**

Gene Ontology (GO) annotation proteome was derived from the UniProt-GOA database (www. http://www.ebi.ac.uk/GOA/). The proteins were classified by GO annotation based on three categories: biological process, cellular component and molecular function. Proteins domain functional description was annotated by InterProScan based on protein sequence alignment method, and the InterPro domain database was used. Kyoto Encyclopedia of Genes and Genomes (KEGG) database was used to annotate protein pathway. Wolfpsort (one subcellular localization predication software) was selected to perform the subcellular localization analysis.

**Functional Enrichment Analysis.**

Proteins were classified by GO, KEGG pathway and InterPro domains annotation into different categories. For each category, a two-tailed Fisher’s exact test was employed to test the enrichment of the differentially expressed protein against all identified proteins in house perl script. The enrichment analysis for the three categories in GO-biological process, molecular function and cellular compartment, were performed separately. Correction for multiple hypothesis testing was carried out using standard false discovery rate control methods. The category with a corrected p value <0.05 is considered significant.

**Motif Analysis.**

Soft motif-x was used to analyze the model of sequences constituted with amino acids in specific positions of modifier-21-mers (10 amino acids upstream and downstream of the site) in all protein sequences. And all the database protein sequences were used as background database parameter, central character was specified as modified amino acid K (Lysine), 21 amino acids for peptide length, 20 for occurrences, significance was set at 0.000001.

**Enrichment-based Clustering.**

All the protein categories obtained after enrichment were collated along with their p values, and then filtered for those categories which were at least enriched in one of the clusters with p value<0.05. This filtered p value matrix was transformed by the function x = −log10 (p value). Finally these x values were z-transformed for each category. These z scores were then clustered by one-way hierarchical clustering (Euclidean distance, average linkage clustering) in Genesis. Cluster membership was visualized by a heat map using the “heatmap.2” function from the “gplots” R-package.

**Cluster Analysis of Protein Express Profiles.**

Firstly, combine all the protein quantitative ratios of different samples by protein ID. Then the normalized ratios for the sample profiles of regulated protein were log2 transformed and then normalized so that, for each profile, the mean was zero and standard deviation was one. The normalization of data ensures that proteins with similar temporal patterns are close in Euclidean space. The transformed profiles were then clustered using the Mfuzz toolbox, which is based on the open-source statistical language R. We used the fuzzy c-means (FCM) clustering algorithm, which is part of the toolbox. FCM clustering is a soft parting clustering method that requires two main parameters (c = number of clusters, m = fuzzification parameter) and uses Euclidean distance as the distance metric. FCM assigns to each profile a membership value in the range [0, 1] for each of the c cluster. The algorithm iteratively assigns the profile to the cluster with the nearest cluster center while minimizing an objective function. Parameter m plays an important role in deriving robust clusters that are not greatly influenced by noise and random artifacts in data. For our analysis, the optimal values of c and m were derived. Final clustering was done with the parameters c = 15 and m = 2.

**Protein-protein co-expressed network analyses of Ksucc with lncRNAs.**

Co-expression detection analysis was conducted using the WGCNA R package with soft power 16. For each relationship, there were two values called “Co-expression score” and “Pearson Correlation Coefficient” to assess degree of association. As “Co-expression” produced by WGCNA, the higher values of 0.50 in our study suggest the higher association degree. As “Pearson’s correlation”, the value close to 1 indicates positively regulated correlation. On the contrary, the value close to -1 indicates negatively regulated correlation. The soft Cytoscape (version 3.3.0) was used to visualize network.

**Microarray Assay.**

Briefly, total RNA was isolated from the four pairs of GC specimens using TRizol reagent (Invitrogen, CA, USA) according to manufacturer’s instructions. Total RNA quality and quantity was measured by using Nanodrop spectrophotometer (ND-1000, Nanodrop Technologies) and RNA integrity was determined by standard denaturing agarose gel electrophoresis for both lncRNAs.

For lncRNAs microarray analysis, mRNA was purified from total RNA after removal of rRNA (mRNA-ONLY™ Eukaryotic mRNA Isolation Kit, Epicentre). Then, each sample was amplified and transcribed into fluorescent cRNA along the entire length of the transcripts without 3’ bias utilizing a random priming method. The labeled cRNAs were hybridized onto the Human LncRNA Array v3.0 (8 x 60K, Arraystar). After washing, the arrays were scanned by the Agilent Scanner G2505C. Agilent Fe ature Extraction software (version 11.0.1.1) was used to analyze acquired array images. Quantile normalization and subsequent data processing were performed with using the GeneSpring GX v11.5.1 software package (Agilent Technologies).

**Supplementary Table Legends**

**Supplementary Table 1.** A summary of the clinical and pathological data of four GC patients.

**Supplementary Table 2.** Identification and functional analysis of global proteome in GC.

**Supplementary Table 3.** The dynamic clusters analysis of protein expression profile in GC.

**Supplementary Table 4.** Identification and functional analysis of global lysine succinylome in GC.

**Supplementary Table 5.** Proteins and Ksucc expression levels in TCA cycle and pentose phosphate pathway.

**Supplementary Table 6.** Co-expression and protein-protein interactions analysis reveal the potential crosstalks between lncRNAs and Ksucc in GC.

**Supplementary Figure Legends**

**Supplementary Figure 1. Global proteome data generation quality control.** Four pairs of tumor and normal GC tissues were analyzed in two TMT-6 plex experiments (one experiment with 3 pairs of samples and the other with one pair sample). (**a**) Distribution of tryptic length detected by MS/MS from two experiments. Of the two experiments present a similar distribution in peptide length, but different in total number of identified peptide (that may be caused by different sample size in two experiments). (**b**) Distribution of sequence coverage of the identified proteins with tryptic peptides detected by MS/MS of two experiments. (**c**) Protein quantitation distribution of four pairs of samples. Quantitation of four pairs sample presents a similar normal distribution that consistent with normal physiological dynamic changes.

**Supplementary Figure 2. The heat map of global protein expression and the pearson's coefficient in four pairs of gastric cancer.** (**a**) Global protein expression levels in these samples. (**b**) The pearson's coefficient of these samples revealed similar global protein expression profile.

**Supplementary Figure 3. Enrichment-based dynamic cluster analyses of protein expression profile in gastric cancer.** We have concluded a total of 15 dynamic clusters analysis in the process of LNM. The abscissa represents the samples and the ordinate represents the quantitative values of each sample in log2 conversion.

**Supplementary Figure 4. Full-length and multiple exposures gels of global lysine succinylation** **are marked with red rectangles and molecular sizes indicated in kDa.** (**a**) The expression profile of global lysine succinylation in GC tissues by western blotting using with a pan anti-succinyl lysine polyclonal antibody for long-time exposures. (**b**) The expression profile of global lysine succinylation for short-time exposures. (**c**) The expression profile of GAPDH for long-time exposures. (**d**) The expression profile of GAPDH for short-time exposures.

**Supplementary Figure 5.** **Lysine succinylation data generation quality control.** Four pairs of tumor and normal GC tissues were analyzed in two TMT-6 plex experiments (one experiment with 3 pairs of samples and the other with one pair sample). (**a, b**) Distribution of succinylation sites per peptide with different length in two experiments. This graph showed a high efficiency identification with succinylated peptides in two experiments. There were 92% (**a**) and 89% (**b**) peptides with at least one succinylation site detected. (**c, d**) Distribution of succinylation sites per peptide which identified in different fragment. This graph showed a uniform distribution of succinylated peptides in each fragment. (**e**) Distribution of lysine succinylation sites quantitation of four pairs of samples. Quantitation of four pairs sample presents a similar normal distribution that consistent with normal physiological dynamic changes.

**Supplementary Figure 6. Functional analysis of lysine succinylome in gastric cancer.** (**a**) The subcellular localization of succinylated proteins. GO functional annotation (level 2) including (**b**) biological process, (**c**) cellular component and (**d**) molecular function.

**Supplementary Figure 7. The expression of PCNA in the remaining 16 pairs and full-length gels of PCNA.** (**a**) The expression level of PCNA was also up-regulated in cancer tissues compared with their matched normal ones in the remaining 16 pairs of GC tissues. (**b**) Full-length gels of PCNA and GAPDH.

**Supplementary Figure 8.** **Full-length gels of Ksucc-K569 of CALD1.**

**Supplementary Figure 9.** **The workflow and strategy for searching crosstalks between lncRNAs and Ksucc by co-expression detection and protein-protein interactions analysis in gastric cancer.**

**Supplementary Figure 1.**

**
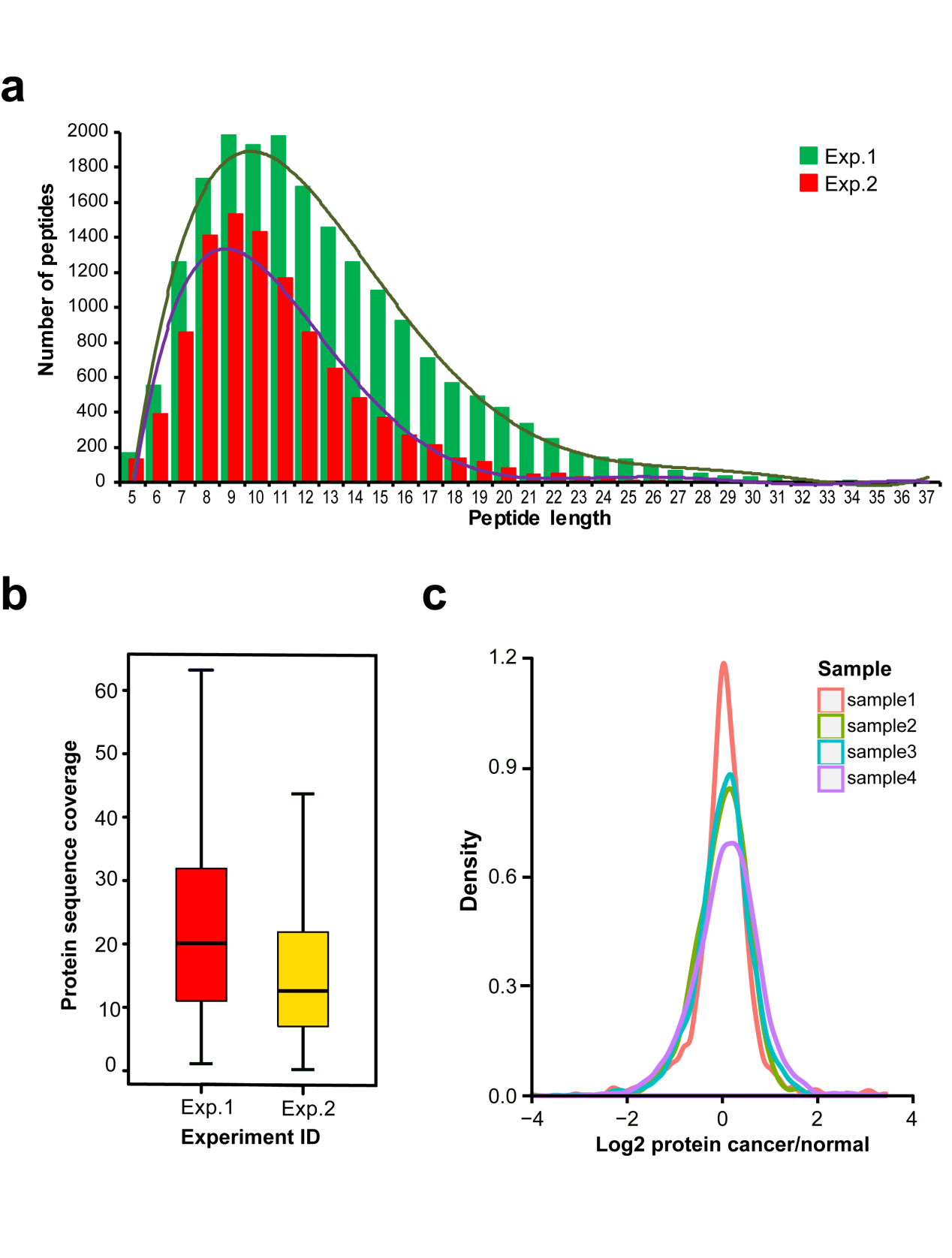
**

**Supplementary Figure 2.**

**
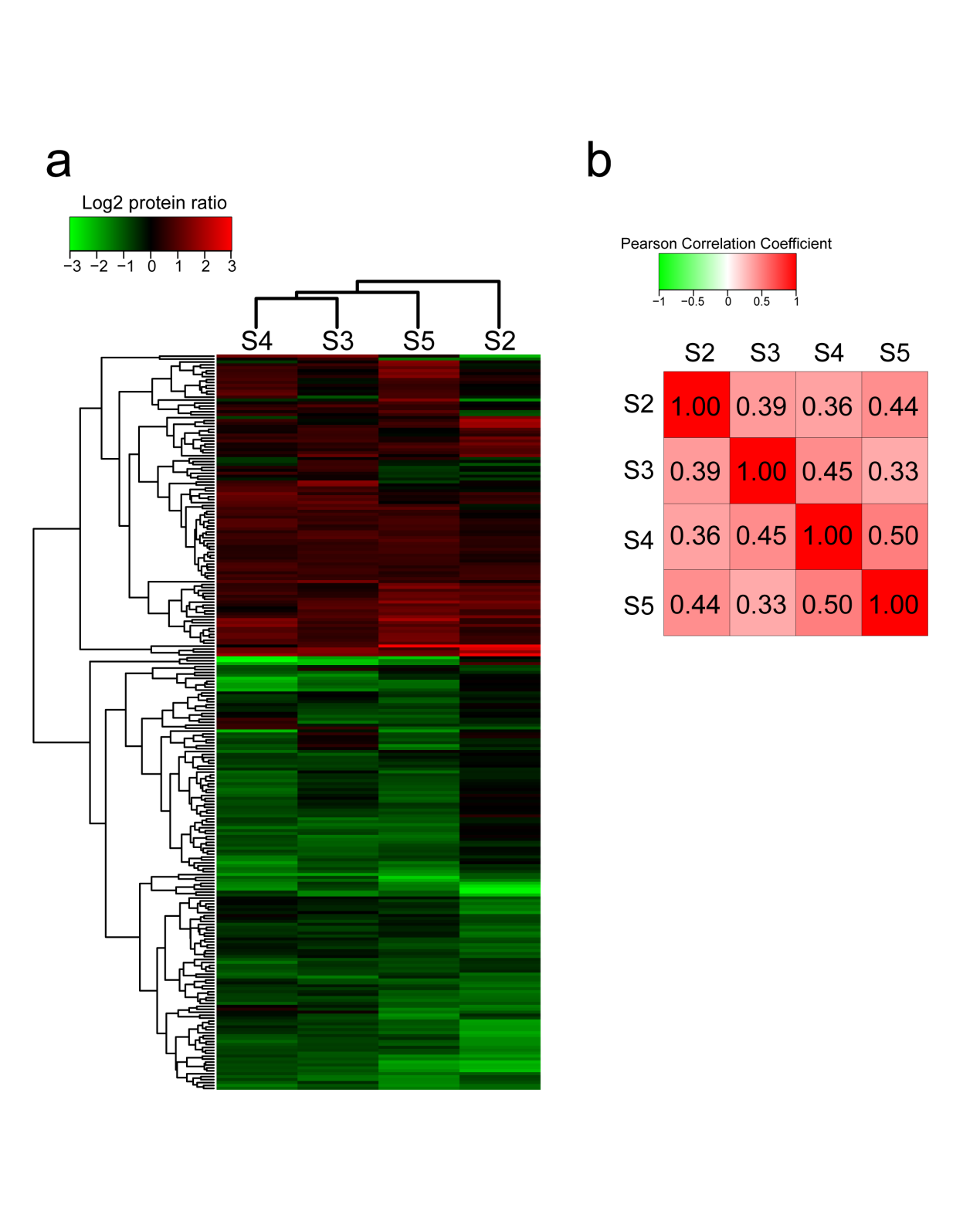
**

**Supplementary Figure 3.**

**
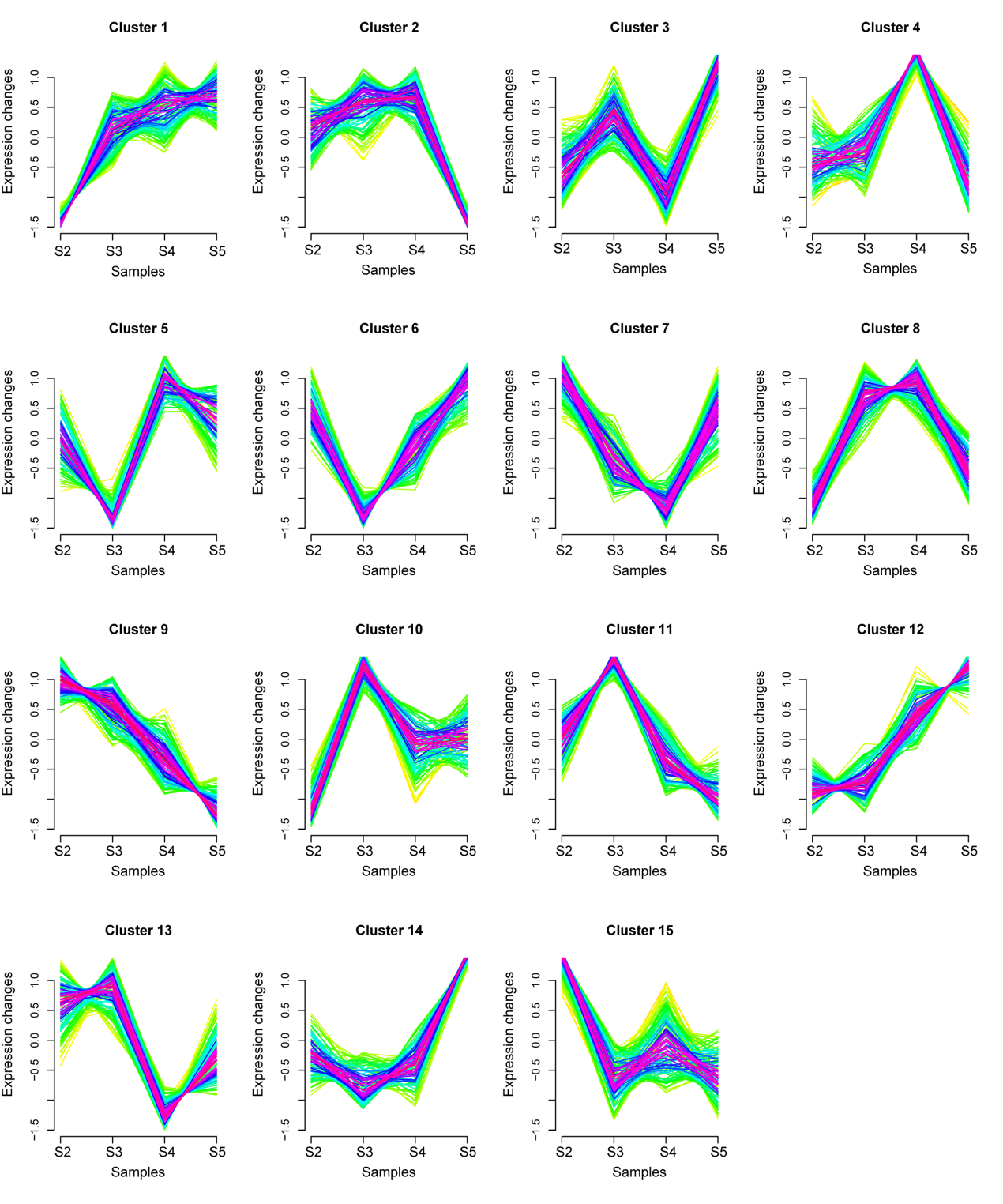
**

**Supplementary Figure 4.**

**
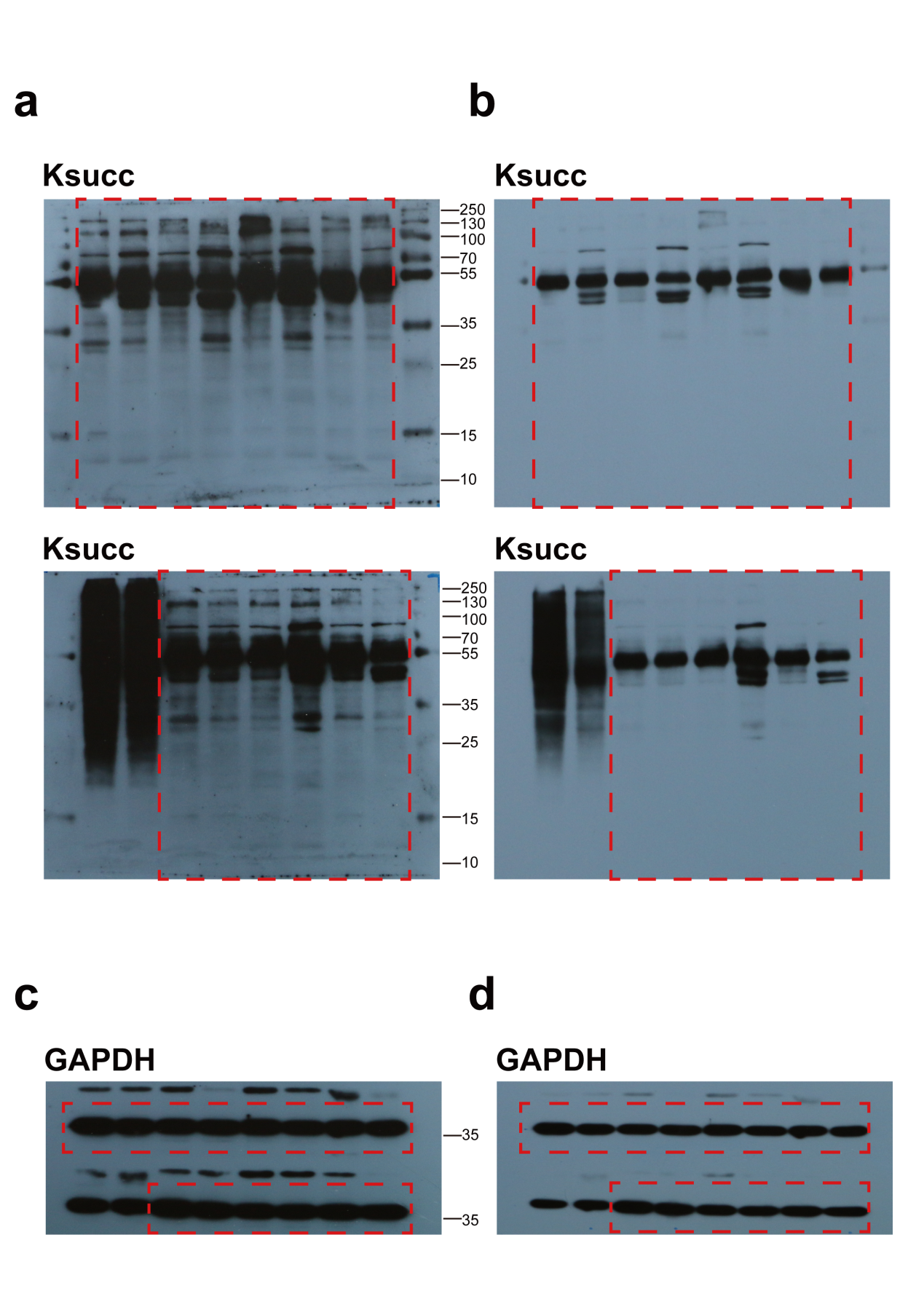
**

**Supplementary Figure 5.**

**
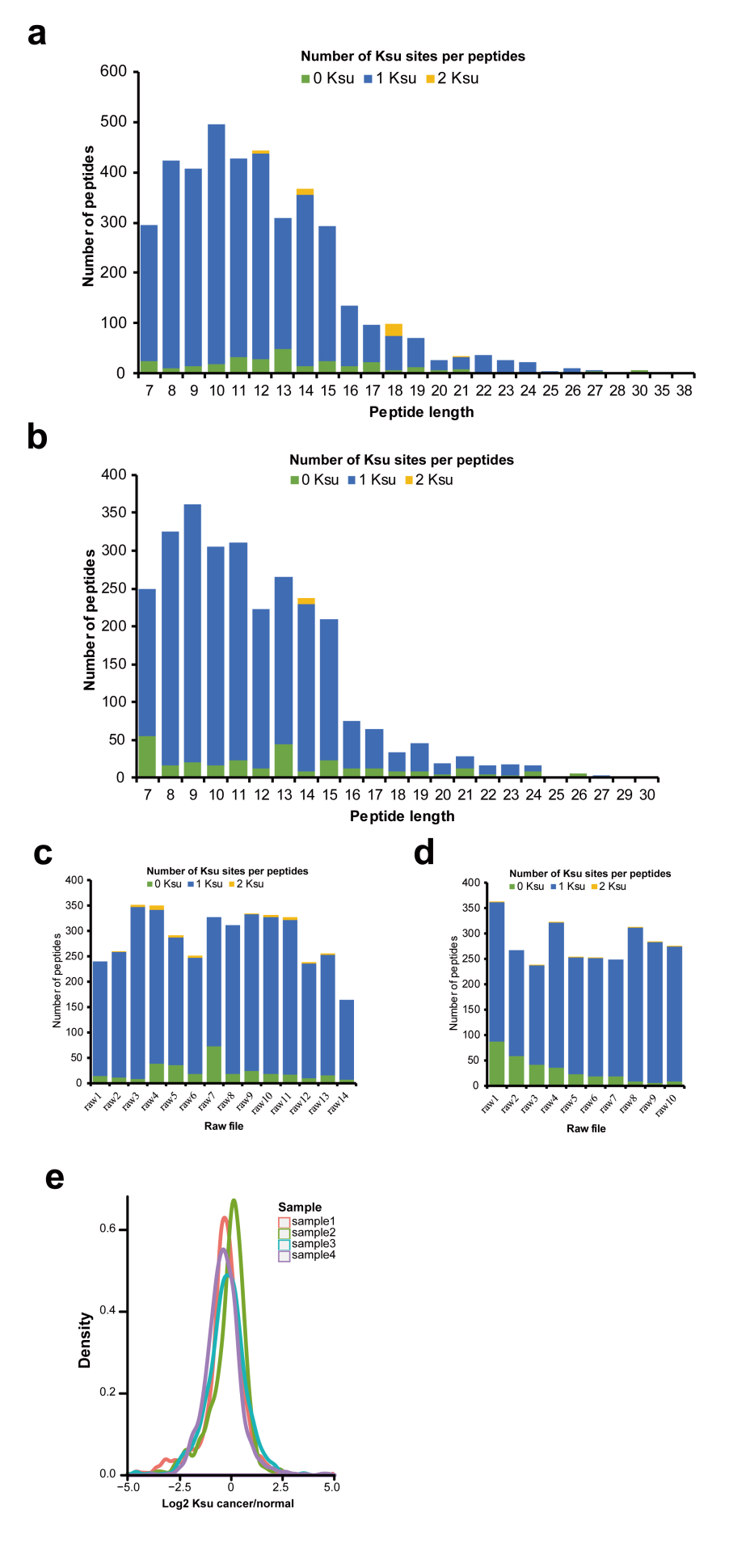
**

**Supplementary Figure 6.**

**
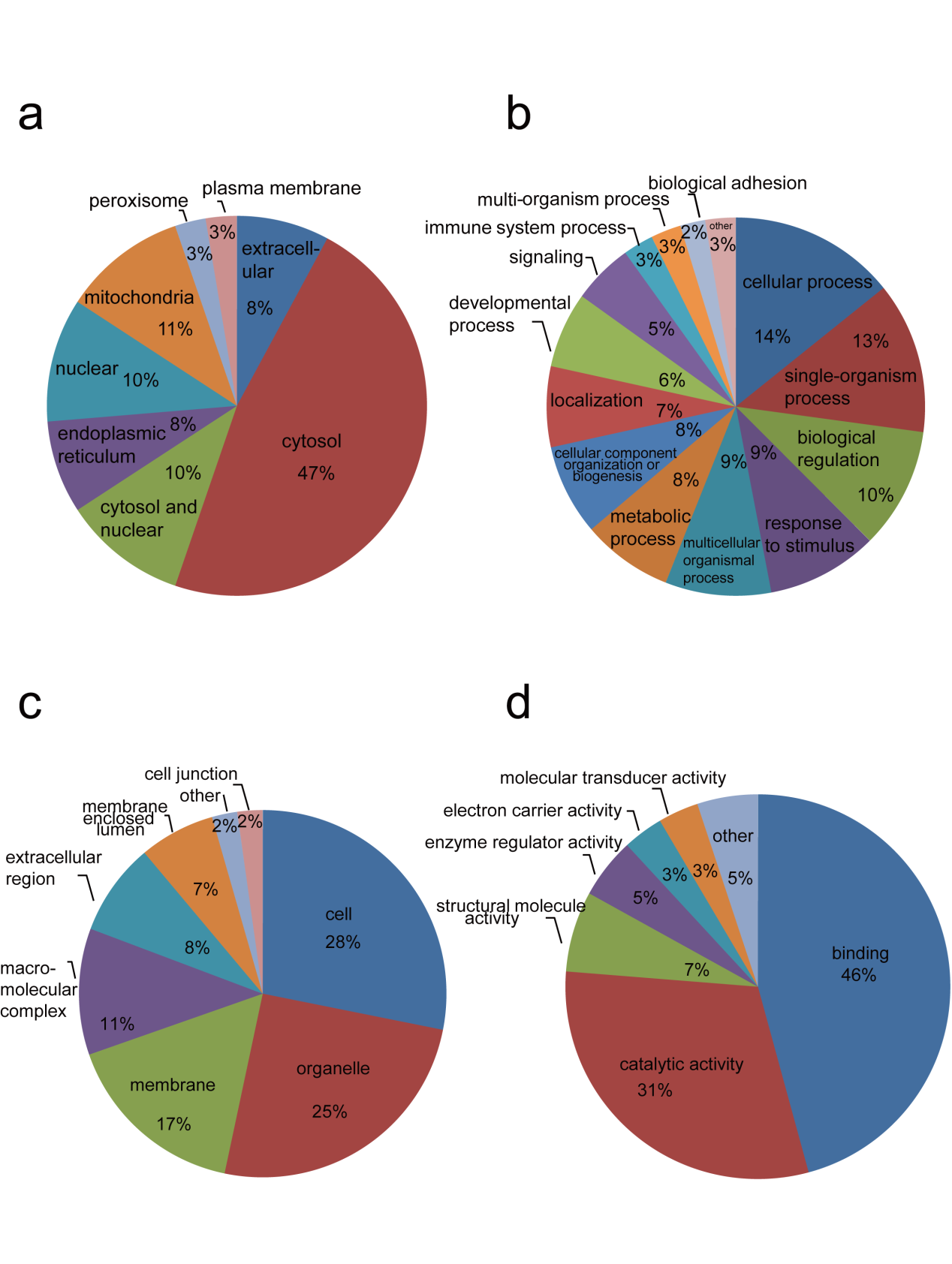
**

**Supplementary Figure 7.**

**
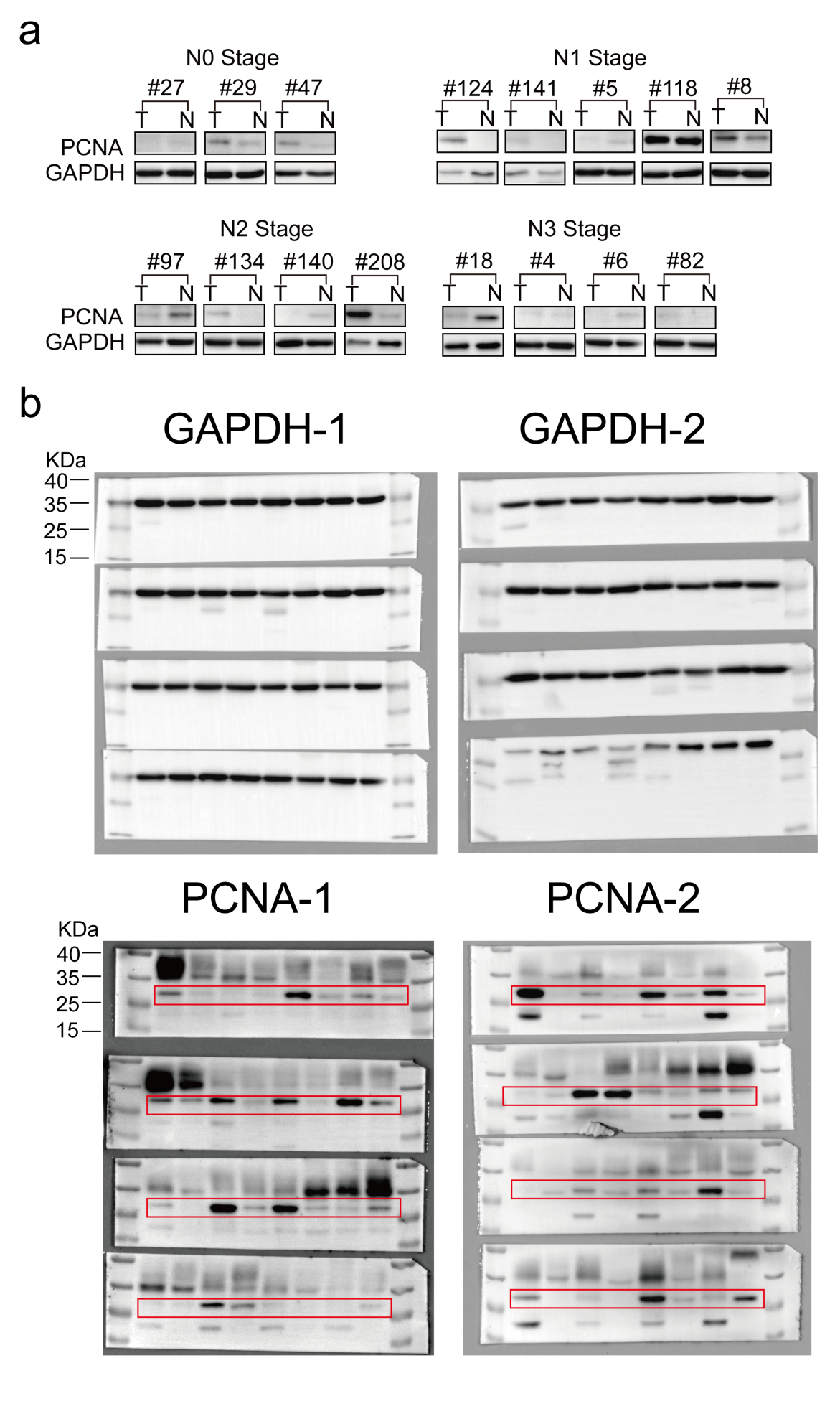
**

**Supplementary Figure 8-1.**


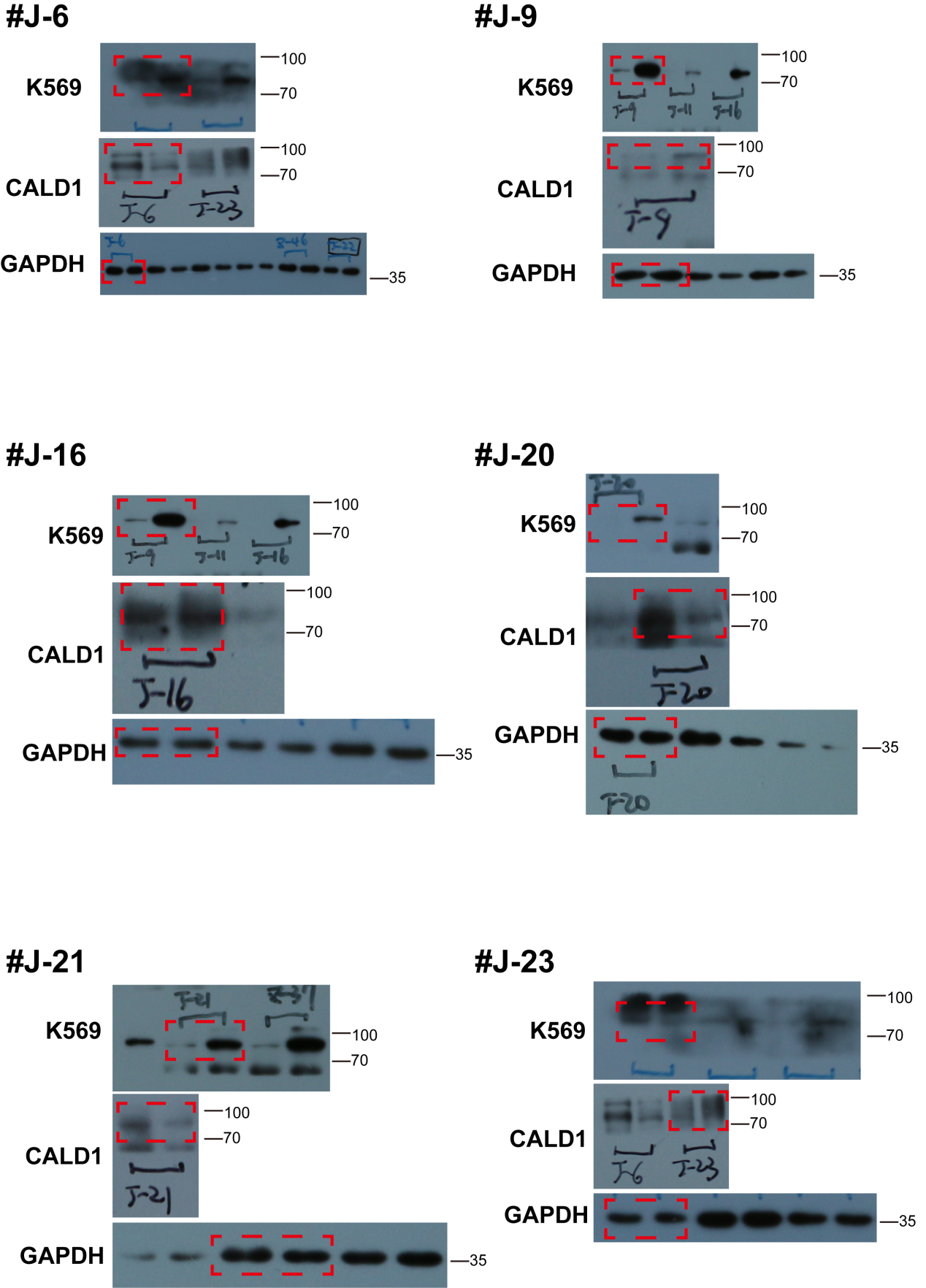


**Supplementary Figure 8-2.**


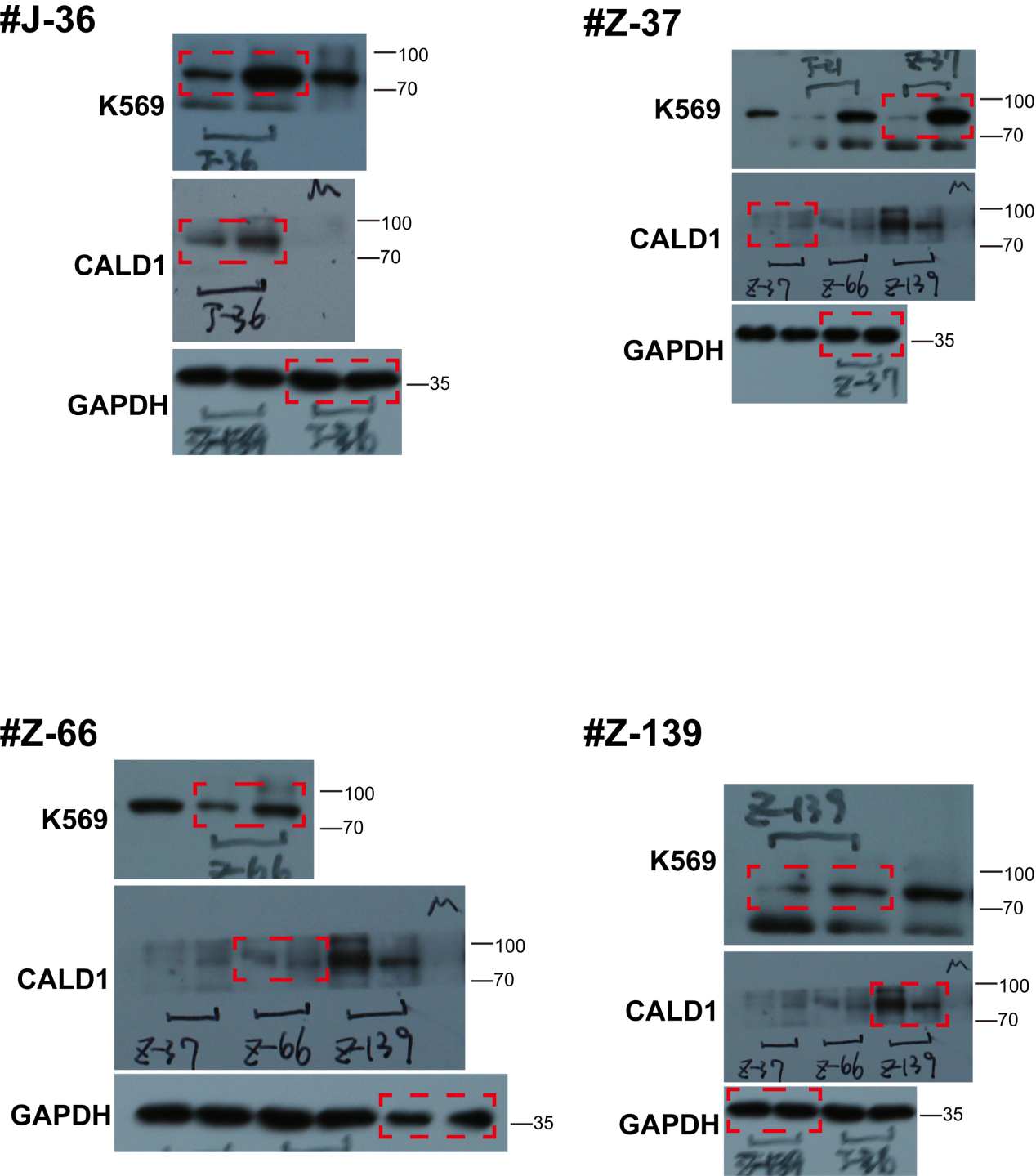


**Supplementary Figure 9.**


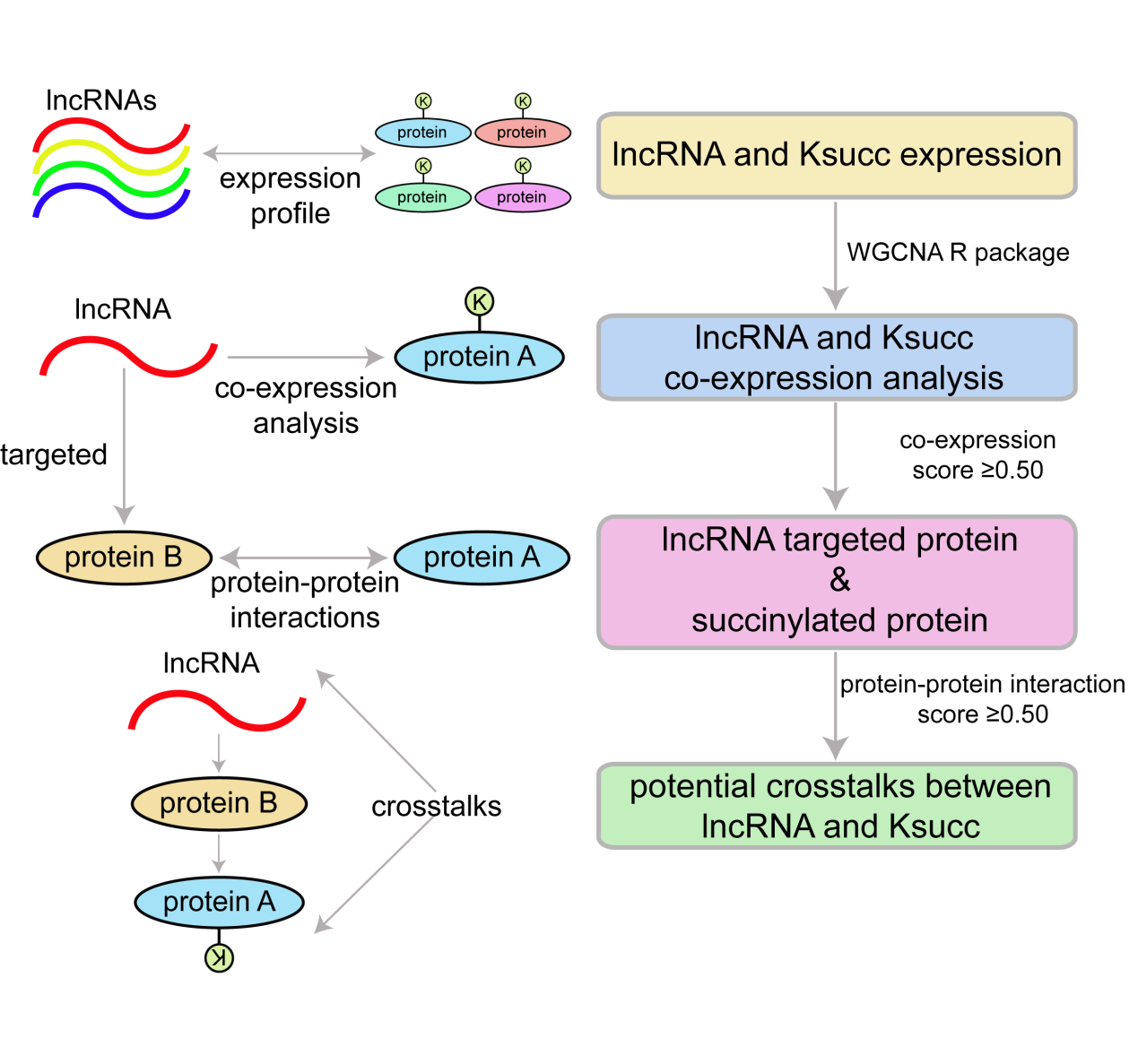

Supplement: Supplementary Information [file srep42053-s1.doc]
